# Experimental Investigation of Four-Point Bending Test Results of GFRP and CFRP Composites Used in Wind Turbine Blades

**DOI:** 10.3390/polym17172412

**Published:** 2025-09-05

**Authors:** Senai Yalçinkaya, Mehmet Fatih Yoldaş, Dudu Mertgenç Yoldaş

**Affiliations:** 1 Faculty of Technology, Department of Mechanical Engineering, Marmara University, Maltepe 34854, Istanbul, Turkey; syalcinkaya@marmara.edu.tr; 2 Mechanical Engineering, Dokuz Eylul University, Buca 35360, Izmir, Turkey; 3 Department of Mechanical and Metal Technologies, Dokuz Eylul University Izmir Vocational School, Buca 35360, Izmir, Turkey; dudu.yoldas@deu.edu.tr

**Keywords:** adhesively bonded joints, composite materials, elastic modulus, four-point bending test, mechanical properties

## Abstract

The depletion of fossil fuels and the rise of environmental concerns have increased the importance of renewable energy sources, positioning wind energy as a key alternative. Modern wind turbine blades are predominantly manufactured from composite materials due to their light weight, high strength, and resistance to corrosion. In offshore applications, approximately 95% of the composite content is glass fiber-reinforced polymer (GFRP), while the remaining 5% is carbon fiber-reinforced polymer (CFRP). GFRP is favored for its low cost and fatigue resistance, whereas CFRP offers superior strength and stiffness but is limited by high production costs. This study investigates the durability of adhesively bonded GFRP and CFRP joints under marine exposure. Seven-layer GFRP and eight-layer CFRP laminates were produced using a 90° unidirectional twill weave and prepared in accordance with ASTM D5868-01. Specimens were immersed in natural Aegean Sea water (21 °C, salinity 3.3–3.7%) for 1, 2, and 3 months. Measurements revealed that GFRP absorbed significantly more moisture (1.02%, 2.97%, 3.78%) than CFRP (0.49%, 0.76%, 0.91%). Four-point bending tests conducted according to ASTM D790 showed reductions in Young’s modulus of up to 9.45% for GFRP and 3.48% for CFRP. Scanning electron microscopy (SEM) confirmed that moisture-induced degradation was more severe in GFRP joints compared to CFRP. These findings highlight the critical role of environmental exposure in the mechanical performance of marine composite joints.

## 1. Introduction

Polymer matrix composite materials are widely used in offshore wind energy systems and marine applications due to their high specific strength, corrosion resistance, and long-lasting structures [1,2]. However, marine environmental conditions can lead to physical, chemical, and mechanical changes in these materials, such as water absorption, aging, loss of durability, and microcrack formation, upon long-term exposure [3]. Therefore, water absorption behavior, the resulting damage progression, and durability loss mechanisms in polymer composites are of critical importance.

Water absorption in composite materials is evaluated within the framework of three main mechanisms. The first is the diffusion of water molecules within the amorphous regions of the polymer matrix, and this behavior is often explained in accordance with Fick’s diffusion law [4]. In this case, moisture absorption occurs rapidly initially, and an equilibrium concentration is reached over time. However, due to the relaxation of polymer chains, the formation of microcracks within the matrix, or structural heterogeneities, water transport can often exhibit non-Fickian diffusion characteristics [5,6]. The second mechanism is the movement of water through capillary effects at the fiber-matrix interfaces and microvoids [7]. This process plays a particularly important role in composites with a high concentration of voids and defects formed during production. The third mechanism is related to the hydrophilic or hydrophobic nature of the fiber; while GFRP exhibits high moisture absorption, this rate is lower in CFRP [8,9]. Following water absorption, a series of durability degradation mechanisms come into play in composites. The diffusion of water molecules into the matrix causes plasticization, a decrease in the elastic modulus, and a loss of mechanical stiffness [10]. In addition, Na^+^ and Cl^−^ ions present in seawater accelerate hydrolysis reactions within the polymer matrix, causing chain breaks, which weakens the integrity of the matrix [11]. In addition, the diffusion of water to the fiber-matrix interface weakens adhesion forces, negatively affecting load transfer and the durability of the joint [4]. Especially in glass fibers, ionic dissolution and corrosion processes accelerate microcrack propagation, thus creating a cycle that accelerates the aging process of the composite [3,7]. These interface weakenings accelerate the formation and propagation of microcracks, and the growth of cracks paves the way for water to penetrate deeper into the composite [10]. As a result, polymer composites exposed to seawater experience a loss of mechanical performance over time, including decreased durability, decreased flexural and tensile strength, and decreased fatigue life [1,4,10]. Microcrack formation and interface separation limit the long-term use of the material. Therefore, material selection and design strategies should be developed considering aging mechanisms, water absorption behavior, strength losses, and damage progression during the use of polymer composites in marine environments [2,11]. Significant decreases in tensile strength, modulus of elasticity, flexural strength, and fatigue life have been reported in the literature for polymer composites exposed to seawater [3,4,5,6,7,8,9,10]. Single-lap adhesive joints used in polymer matrix composites are widely preferred, particularly in engineering structures requiring lightweight and high load-bearing capacity. This joint type is highly sensitive to both adhesive properties and joint geometry because load transfer occurs through the adhesive layer [12]. Load transfer in single-lap joints occurs primarily through shear and peel stresses. However, the limited overlap length and the axial transfer of load lead to stress concentrations at the joint end regions [13]. Therefore, during long-term use in marine environments, the diffusion of water into the adhesive layer and the resulting bond weakening at the interface become a critical mechanism that directly affects joint life [14]. Studies have shown that the strength of single-lap adhesive joints is significantly affected by parameters such as overlap length, adhesive thickness, surface preparation, and environmental conditions [15]. For example, although increasing the overlap length improves load transfer, it provides no additional advantage after a certain length and can even lead to increased peel stresses [16]. Similarly, increasing the adhesive layer thickness can reduce long-term strength by facilitating water diffusion [17].

In experimental studies on adhesive joints exposed to seawater, significant decreases in mechanical properties were observed as the adhesive layer absorbed moisture [18]. This is particularly critical for epoxy-based adhesives, and losses in strength have been reported in connection with the accelerated diffusion process. In this context, both the adhesive material selection and surface preparation methods are decisive in extending the joint life [19].

In conclusion, single-lap adhesive joints are widely preferred in composite structures used in marine environments and offshore wind turbine blades due to their light weight, ease of assembly, and load-carrying capacity. However, in order to ensure the reliability of these connections, both material selection and connection design parameters must be implemented considering long-term performance in seawater environmental conditions.

Some of the studies conducted are as follows;

Mertgenç Yoldaş and Yoldaş (2025) investigated the mechanical performance of glass fiber-reinforced (GFRP) and carbon fiber-reinforced (CFRP) composites after exposure to seawater. Specimens were immersed in seawater at 3.3–3.7% salinity and 23.5 °C for 1 to 15 months and subjected to axial impact tests according to ASTM D5868-01. Moisture absorption and fracture behavior were compared in specimens tested under 30 J energy. Interface structures were examined in detail using SEM analyses. After 15 months, an increase in fracture toughness of GFRP was observed to 27.96%, while that of CFRP was observed to 11.96%. Despite exposure to humidity, CFRP specimens generally showed superior mechanical performance. This demonstrates that CFRP materials are more resistant to moisture-induced physical and structural weakening compared to GFRP and exhibit better performance in mechanical properties, particularly flexural strength [19].

Yalçınkaya et al. (2025) investigated the mechanical behavior of GFRP and CFRP composites after exposure to seawater for marine applications. Specimens were immersed in Aegean Sea water for 30 and 60 days, and then subjected to moisture retention, Charpy impact, and three-point bending tests. The results showed that CFRP specimens exhibited lower moisture absorption and a more stable modulus of elasticity than GFRP. GFRP absorbed more energy but exhibited more brittle behavior. Overall, CFRP materials are superior to GFRP materials in terms of mechanical performance. CFRP has demonstrated superior results, particularly in terms of durability, strength, and resistance to deformation. This superior performance is one of the main reasons why CFRP is preferred in structural applications [20].

Ji and Han (2014) investigated the crack behavior in adhesively bonded wind turbine blades using the cohesive zone model (CZM) and the finite element method (FEM). The study determined that high shear stresses, in particular, initiated damage at the joint line, initiating this damage from the joint edges and leading to progressive crack growth under load. Modeling results were also supported by experimental findings, emphasizing the determinant role of adhesive thickness and material compatibility in damage behavior [21].

Khoshmanesh et al. (2023) investigated the development of a method for local damage detection in spar cover and shear web joints with thick adhesive layers. In this study, phase changes in mode shapes were observed due to microdamage occurring in the joint area, and this change provided important clues about the location and magnitude of damage. This study, conducted under fatigue tests, demonstrated that mode shape phase analysis can detect damage at an early stage [22].

Rosemeier et al. (2019) investigated the sensitivity of adhesively bonded joints not only to mechanical loading but also to residual stresses and environmental influences originating from the manufacturing process. It was shown that differences in thermal expansion during the manufacturing process caused tunneling cracks in the joint area. These internal stresses, rather than the mechanical load, facilitated crack propagation over time and reduced the life of the structure. Moisture penetration has also been cited as a factor accelerating the propagation of these cracks [23].

Rafiee and Hashemi-Taheri (2021) analyzed the effects of adhesive thickness and tape width on the performance of trailing edge joints using three-dimensional finite elements and cohesive zone models (CZM). The results showed that selecting the appropriate adhesive thickness can significantly improve joint strength and prevent damage propagation [24].

Four-point bending tests are widely used to evaluate the bending behavior of composite materials used in wind turbine blades.

In this regard,

Musial et al. (2001) investigated a study conducted by the US National Renewable Energy Laboratory (NREL). In a study conducted by the US National Renewable Energy Laboratory (NREL), pultrusion-produced glass fiber-reinforced composite panels were subjected to full-scale four-point bending tests. 2.4 m-long panel specimens were tested in different configurations, receiving loads from both high-pressure and low-pressure surfaces. As a result of the study, early local buckling was observed, especially on high-pressure surfaces; however, it was determined that the structures continued to carry loads for some time after buckling. This shows that buckling behavior is not always a strength criterion and that the load-carrying capacity of the structure can be maintained through alternative load paths [25].

Maldonado-Santiago et al. (2023) investigated the behavior of composite box-beam profiles representing a wind turbine blade structure under four-point bending. This study, which used three different profile designs, tested two different configurations, including inverted V-shaped and X-shaped stiffeners, in addition to the basic profile. Four-point bending tests revealed that the stiffening structures increased stiffness and significantly reduced displacement. In the inverted V-shaped profile, stiffness increased by 43.41%, while maximum displacement decreased by 30%. Furthermore, modeling studies using finite element analysis showed high agreement with experimental data, indicating that the maximum deflection remained at 11%. When examining the fracture modes, matrix cracking, laminate separation, and local fractures at the joints were observed [26].

Mishnaevsky et al. (2023) investigated the use of biomimetic design principles and engineering adhesives to increase the structural durability of wind turbine blades. The study emphasized that the most common failure mechanisms in wind turbine blades are largely due to weak and vulnerable interfaces and adhesive zones. In this context, the mechanical strength and long-term reliability of adhesive materials are critical for the sustainability of blade performance. They demonstrated that a biomimetic design approach can be applied to address this issue and increase structural integrity, demonstrating that optimizing adhesives can play a decisive role in blade life and performance. Thus, they presented a new perspective on both material selection and joint and interface design in wind turbine blade design [27].

Arwood et al. (2025) investigated the mechanical behavior of CFRP panels produced by pultrusion method in detail through four-point bending tests. The test method used in the study aimed to evaluate the strain distribution to which the panels were subjected under large-span loading conditions. During the experimental process, strain changes on the surface of the panels were monitored using high-resolution fiber optic sensor technology. The obtained data revealed that the four-point bending test allows for detailed analysis of the stress fields and material responses, especially those occurring in the loading zone [28].

Existing research has not adequately examined the long-term effects of natural seawater conditions on adhesively bonded FRP composite joints used in offshore wind turbine blades. Most studies have focused on synthetic brine or short-term environmental exposure, thus ignoring the realistic damage processes induced by the combined effects of temperature and salinity. Furthermore, the no-slip load transfer behavior of the adhesive layer under pure bending has not been directly evaluated. While the mechanical performance of composite materials has been frequently studied, simultaneous and holistic analysis of the stress distribution, damage initiation, and crack propagation mechanisms in the joint zone has not been adequately conducted.

This study experimentally investigates the damage processes in seawater of FRP composite materials, particularly in adhesively bonded joints, commonly used in offshore wind turbine blades.

Unlike many studies in the literature, this study experimentally addresses the deterioration processes in seawater-induced deterioration of FRP composites, commonly used in wind turbine blades, in offshore conditions. Most previous studies have focused on the general mechanical properties of materials and the performance of adhesives only in dry environments. In contrast, this study systematically and comprehensively investigates the effects of deterioration occurring directly in the marine environment on joint strength using four-point bending tests.

Furthermore, the experiments were conducted under constant moment conditions, allowing the adhesive layer to carry loads without shearing deformation. This allows for a detailed examination of both the stress distribution in the joint areas and the damage propagation mechanisms.

Therefore, the current study examines not only the strength properties of composite materials but also the long-term performance of adhesively bonded joints, thus directly contributing to the development of reliable and optimized blade designs for offshore wind turbines. Thus, the study aims to provide an original and innovative contribution to the blade designs of offshore wind turbines by highlighting the experimental analysis of seawater effects and the critical role of material selection in connection design.

## 2. Materials and Methods

### 2.1. Raw Material

The composite materials used in the study were designed in two different types: GFRP and CFRP. GFRP laminates consist of seven layers, while CFRP laminates consist of eight layers, taking into account volume and density ratio. In both composites, the fiber orientation is arranged at 0/90 degrees, and the weave type is twill weave. The fiber used in the GFRP consists of a glass fiber weft yarn with a density of 390 g/m^2^, while in the CFRP, a 3 K carbon fiber weft yarn with a density of 245 g/m^2^ was used.

Prepreg production was carried out on a drum-type prepreg machine for both composites, and the same resin system was used for both material groups. This system consists of F-RES 21 epoxy resin and F-Hard 22 hardener. The laminate thickness was determined to be 2 mm for both composites. Fibermak Engineering Company (Izmir, Turkey) produces GFRP and CFRP composite sheets.

The selection of F-RES 21 epoxy resin and F-Hard 22 hardener is crucial for achieving the highest interfacial adhesion performance in composite materials. The chemical structure and curing mechanism of these components are designed to form a strong and durable bond with the composite surface. Compatibility of the surface preparation with the composite material enhances adhesion quality. Furthermore, the mechanical properties of F-RES 21 epoxy resin and F-Hard 22 hardener—the balance of elastic modulus, hardness, and ductility—were selected to be compatible with the composite structure. This minimizes stress concentrations and damage at the interface, enhancing mechanical performance and durability. The epoxy-based resin system used in the production of composite materials consists of F-RES 21 epoxy resin and F-Hard 22 hardener. These two components were mixed at a 100:21 weight ratio to obtain a homogeneous mixture. The viscosity of this mixture at 25 °C was measured in the range of 500–800 mPa·s. Following prepreg production, curing was carried out at 120 °C for 60 min to achieve final hardness. The epoxy and hardener mixture was applied to the fiber-reinforced structure using the hand lay-up method, and the prepreg material was left at room temperature for one day to complete the gelation process. The composite material was shaped by hot pressing after the resin gelled and cured for one hour by applying 8–10 bar pressure at 120 °C.

### 2.2. Sample Preparation

**Figure 1:**
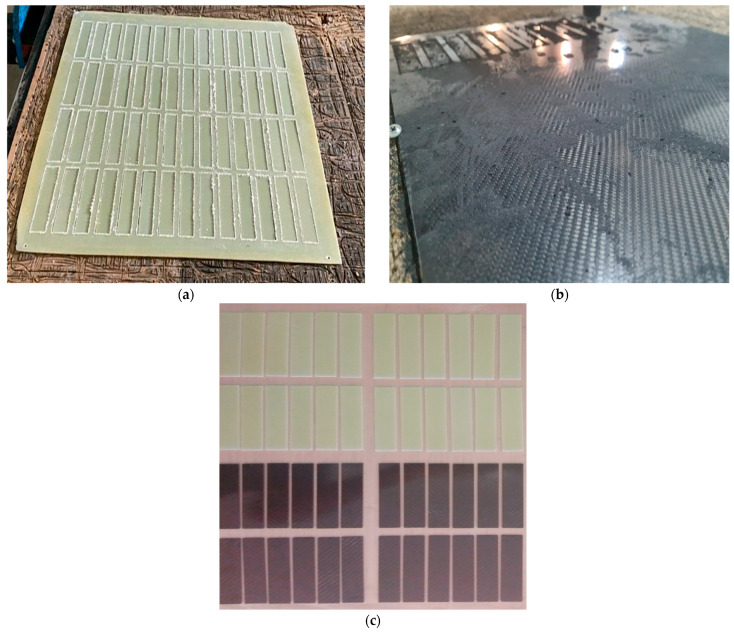
Shaping of GFRP (**a**), CFRP (**b**) Plates with CNC Router and GFRP-CFRP samples subjected to sanding (**c**).

The produced GFRP and CFRP composite sheets were designed with dimensions of 500 mm × 500 mm, and a CNC milling machine was used for precision cutting. After cutting, sanding was applied to minimize surface roughness and increase surface flatness. This process allows for homogeneous contact with the adhesive surface, which directly affects the interface overlap performance. The homogeneous contact area ensures more even load distribution across the adhesive layer, minimizes local stress concentrations, and reduces the risk of premature delamination. Consequently, the applied machining and surface finishing steps support reliable and repeatable measurements of interface strength, increasing the accuracy of the experimental results (Figure 1). After the curing process, the mechanical properties of the composite structures were determined as tensile strength 80 MPa, tensile modulus 3300 MPa, flexural strength 125 MPa and flexural modulus 3200 MPa.

**Figure 2:**
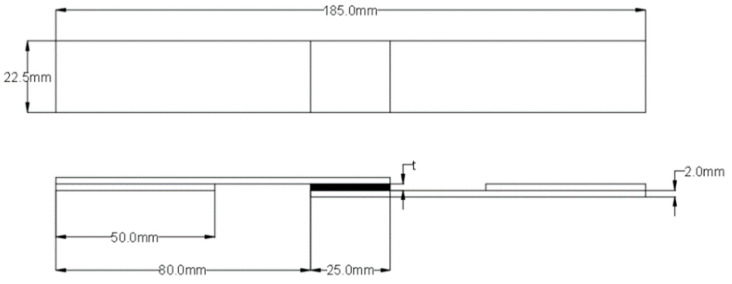
Geometric configuration of GFRP and CFRP specimens.

**Figure 3:**
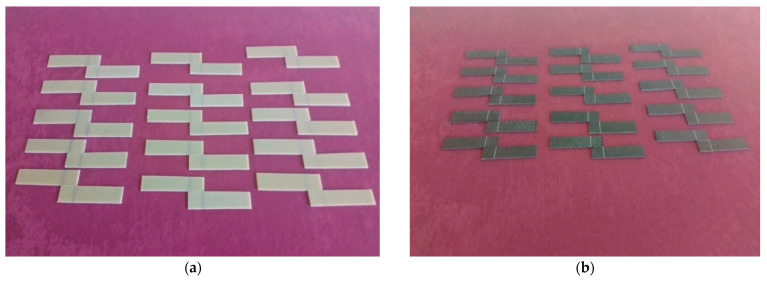
Marking of GFRP (**a**) and CFRP (**b**) specimens for alignment and placement before bonding.

The GFRP and CFRP specimens were prepared in accordance with ASTM D5868-01 [29], the standard test method for evaluating lap shear strength in fiber-reinforced plastic (FRP) adhesively bonded joints (Figure 2). Accordingly, the test specimens were cut to appropriate dimensions and shaped to allow for the examination of both moisture absorption and adhesive bond performance. During the preparation phase for the bonding process, reference points located 25 mm from the ends of the GFRP and CFRP specimens were determined, and the corresponding markings were made (Figure 3).

**Figure 4:**
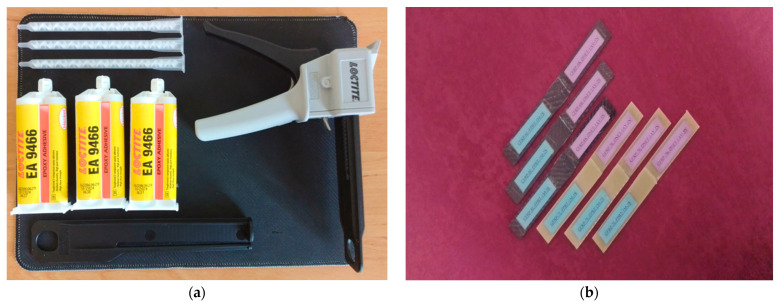
General appearance of the surface after application of Loctite Hysol-9466 epoxy adhesive (**a**) on the samples (**b**).

The samples were prepared by bonding solvent-cleaned surfaces using the adhesive bonding method. The bonding was performed using Loctite Hysol-9466 (Alpanhidrolik, Eskişehir, Turkey), a two-component epoxy adhesive that cures at room temperature and is mixed at a 2:1 ratio in the application gun (Figure 4a). Literature indicates that maintaining the adhesive thickness between 0.1 and 0.3 mm provides optimum bond strength, while thicknesses exceeding 0.6 mm result in a decrease in mechanical strength. This is explained by the fact that thin adhesive layers can utilize mechanical strength more efficiently [30]. Therefore, the adhesive layer thickness was set at 0.2 mm, and application was carried out under a constant pressure of 0.1 MPa. The homogeneity of the adhesive layer was checked with a digital caliper. The samples were allowed to cure at room temperature for 7 days in accordance with the product technical data sheet (Figure 4b).

**Figure 5:**
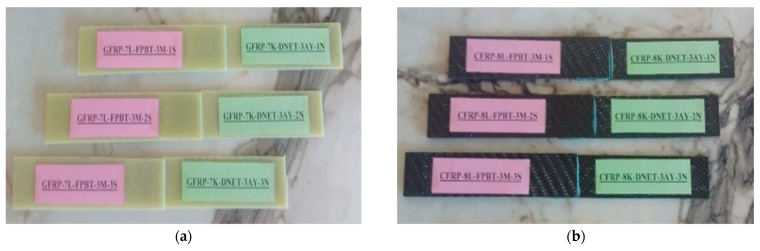
Sample codings of GFRP (**a**) and CFRP (**b**) samples.

To avoid confusion during the experimental process, samples were assigned codes in English. The code structure was organized to include information such as material type, number of layers, test method, environmental conditions, and sample order. For example, the code GFRP-7L-FPBT-2M-1S represents a seven-layer (7L) glass fiber-reinforced plastic (GFRP) sample subjected to four-point bending test (FPBT), immersed in seawater for two months (2M), and the first-row sample (1S) (Figure 5). This coding facilitated accurate identification of samples and collection of experimental data throughout the experiment (Table 1).

**Figure 6:**
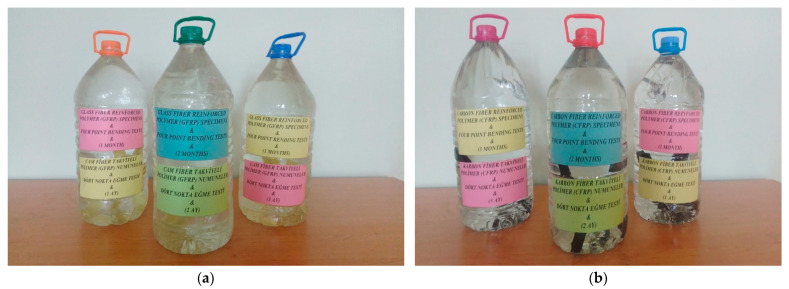
Soaking of GFRP (**a**) and CFRP (**b**) samples in seawater with 3.3–3.7% salinity for 1, 2 and 3 months.

The samples were prepared using the single-lap joint method and divided into three experimental groups for exposure to different environmental conditions. The first group was stored in dry conditions, while the other groups were stored in seawater with a salinity of 3.3–3.7% and a constant temperature of 21 °C for 1, 2, and 3 months, respectively. The salinity and temperature of the seawater to which the samples were exposed were kept constant throughout the experimental period (Figure 6).

### 2.3. Comparative Investigation of GFRP and CFRP Composites Under Different Aging Conditions in Seawater

**Figure 7:**
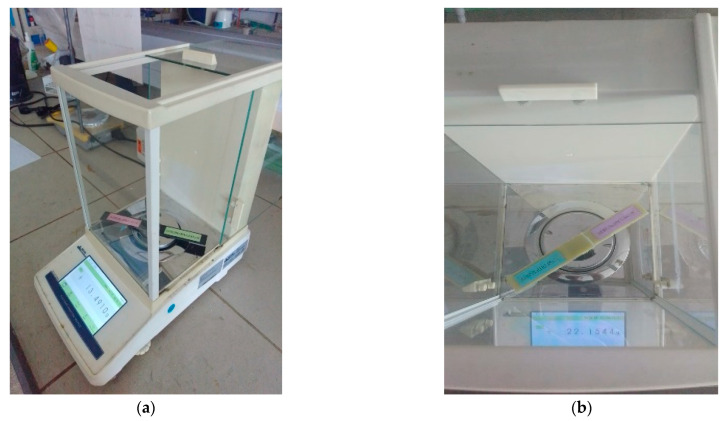
Analytical balance with 0.1 mg precision (DAIHAN Sci. Lab & Med. Inst. Mf, Seoul, Republic of Korea) used to measure sample weights (**a**,**b**).

GFRP and CFRP composite specimens were prepared using the single-layer adhesive bonding method and subjected to aging in seawater conditions. To examine the moisture sorption behavior of the samples, each group of samples was stored in separate containers in seawater with a salinity of 3.3–3.7% and an average temperature of 21 °C for 1, 2, and 3 months. Weight measurements were carried out using an analytical balance with a sensitivity of 0.1 mg at the Dokuz Eylül University Faculty of Science, Daihan Biomedical Chemistry Department Laboratory (DAIHAN Sci. Lab & Med. Inst. Mf, Seoul, Republic of Korea)A total of 12 measurements were made using three standard samples and dry reference samples for each aging period and sample type. Graphs were generated for the average mass changes in the samples based on the obtained data (Figure 7).

### 2.4. Test Method

#### Four Point Bending Test

Because composite structures used in wind turbine blades operate under both aerodynamic and structural loads, reliable assessment of their mechanical performance is crucial. Four-point bending tests are preferred to understand the behavior of blade sections under bending, especially in modern turbines with long blade geometries.

**Figure 8:**
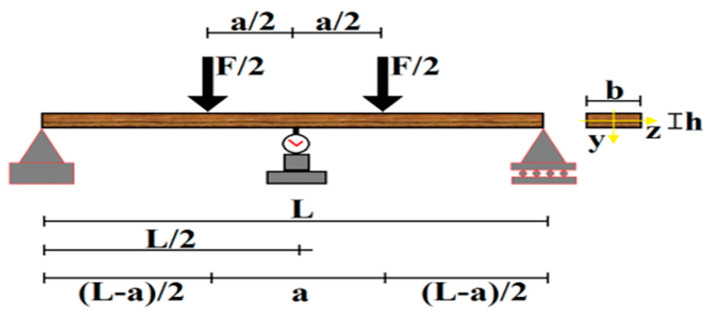
Schematic view of four-point bending test conditions.

The primary reason for choosing four-point bending tests for composite materials is that the material’s anisotropic structure and fiber-matrix interface properties are not adversely affected by local stress concentrations during the measurement process. Compared to three-point bending tests, four-point bending tests offer significant advantages in terms of stress distribution across the sample. Because the load is applied at a single point in a three-point bending test, high local stress concentrations occur in this region, and the sample is at risk of fracture, especially in areas close to the loading point. This can lead to results that do not fully reflect the material’s true elastic and plastic behavior. In four-point bending tests, the load is applied at two points, and a constant bending moment is generated in the interior region between these points. This homogeneous stress distribution, the effect of shear forces is reduced, and a wide area can be examined under pure bending [31]. This feature enables more precise, repeatable, and reliable measurements of mechanical parameters such as flexural modulus and ductility in anisotropic structures such as composite materials. Furthermore, four-point bending tests allow for clearer observation of defects and damage propagation mechanisms in the material. Thus, damage types such as fiber orientation disorders, resin voids or delamination, which are defects specific to composites, can be observed more clearly in this test. The four-point bending test is a test method in which the deformation of a flat test piece, usually of circular or rectangular cross-section, placed freely on two supports is examined by applying two equal forces without changing direction. The region between the load points is defined as the bending zone subjected to a constant bending moment. This reduces the effect of shear stresses, allowing a more accurate assessment of the material’s bending behavior (Figure 8) [32].

In this study, the effects of adhesive type, joint geometry, and composite material type on mechanical behavior were investigated using a four-point bending test. Damage formation and progression in GFRP and CFRP single-lap adhesively bonded joints aged in seawater for offshore wind turbine blades were analyzed, and changes in mechanical properties over time were evaluated. The results demonstrate the strength performance of composite joints used in offshore wind turbine blades. In the four-point bending test, the maximum stress (stress) and strain (ε) values occurring in the center of the adhesively bonded joint specimen were calculated for each load level to evaluate the mechanical behavior of the material.

The following equation calculates the maximum stress in the region between the load application points in the four-point bending test.

(1) $$\sigma =\frac{3F(L-a)}{2bh^{2}}$$

where

*L:* Span between supports, mm
*a:* the distance between the applied forces, mm
*b*: the specimen width, mm
*h*: the specimen thickness, mm
*F*: applied force, N

Strain, ε the value is calculated using the following formula:

(2) $$\varepsilon =\frac{6h\delta (L-a)}{a\left(3L^{2}-4a^{2}\right)}$$

where

ε: strain, mm/mm
*h*: thickness of the specimen, mm
*L*: support span, mm
*a*: half the loading span, mm
δ: deflection at the middle of the span, mm

Four-point bending tests were conducted in accordance with the ASTM D790 standard to investigate the mechanical behavior of composite structures under bending [33]. The experiments were conducted using a 100 kN capacity universal testing machine with a maximum load of 5 kN and a loading rate of 1 mm/min. Parameters such as load, speed, and sample geometry were defined on the machine throughout the experiment, and measurements were automatically recorded using the device software.

**Figure 9:**
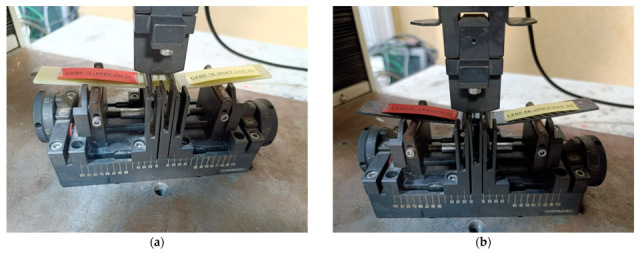
GFRP (**a**) and CFRP (**b**) four-point bending tester and specimen positioning.

GFRP and CFRP single-lap joint specimens with 0.2 mm thick adhesive were used in the study. The specimens were tested in both dry and seawater conditions to evaluate the mechanical performance under environmental conditions. Stress–strain curves were obtained using the test software after the experiment, and analyses were conducted using these data. The effect of environmental effects on the bending behavior at the bond interface was analyzed, and changes in mechanical properties were comparatively examined based on the experimental findings (Figure 9).

A total of 24 connection samples were used, 9 of which were made of GFRP conditioned in seawater conditions, and the other 9 were made of CFRP composite material.

## 3. Results

### 3.1. Moisture Absorption Results

In the CFRP and GFRP single-lap joint specimens, prior to weight measurements, free moisture accumulated on the surface was removed using moisture-absorbing paper to increase measurement accuracy. Following this process, the moisture retention rates of the specimens were calculated using the equation given below, based on the data presented in Table 2 and Table 3.

M(%) = (m_y_ − m_k_)/(m_k_) × (100) (3)

In this equation:

- m_k_: the dry mass of the sample measured before exposure to moisture (g),
- m_y_: wet mass measured after exposure to seawater for a specified period (g),
- M: moisture retention rate of the sample (%) it expresses.

The moisture retention rates of GFRP single-lap joint specimens, which varied depending on the duration of exposure to seawater, were evaluated in comparison with reference specimens stored in a dry environment, which served as a control group (Table 2). The average moisture retention rates of specimens stored in a seawater environment at 21 °C for 1 month, 2 months, and 3 months were determined to be 1.02%, 2.97%, and 3.78%, respectively. These findings suggest that moisture absorption of GFRP specimens increases with increasing seawater exposure time.

Similarly, the moisture retention behavior of CFRP single-lap joint specimens was also examined based on their seawater exposure time and compared with reference specimens stored in a dry environment (Table 3). The average moisture retention rates of CFRP specimens exposed to seawater at 21 °C for 1 month, 2 months, and 3 months were measured as 0.49%, 0.76%, and 0.91%, respectively. The data obtained show that the moisture absorption tendency of CFRP samples increases with exposure time.

**Figure 10:**
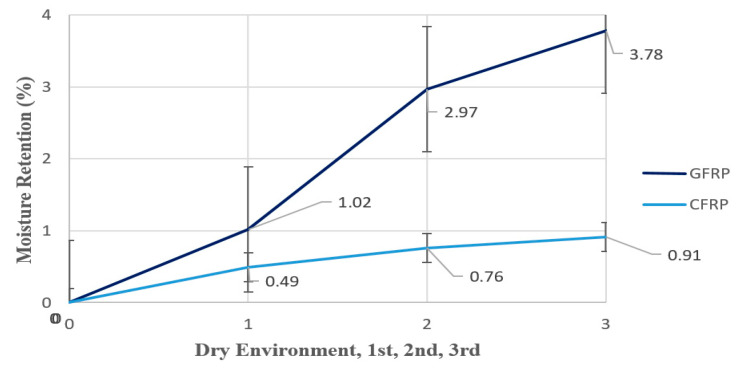
Moisture absorption behavior of GFRP and CFRP connections over time in wind turbine blade connections in offshore conditions.

The comparative moisture absorption behavior of GFRP and CFRP samples is presented in Figure 10.

Figure 10 shows the moisture retention rates (%) of GFRP and CFRP single-lap joint specimens measured after 1, 2, and 3 months of immersion in seawater, representing offshore conditions, respectively. The results show that the GFRP specimens exhibited a tendency toward increasing water absorption over time, and this tendency accelerated, particularly after the second month.

At the end of three months of aging, mass increase continued in GFRP and CFRP. Fick’s theory was used to model moisture diffusion in composite materials. While Fick’s first law assumes a constant diffusion coefficient, Fick’s second law is more appropriate for time-varying environmental conditions and explains the observed changes. According to Fick’s second law of diffusion, the moisture uptake behavior of composite materials is defined as a time-dependent process. When a composite material is exposed to a moist environment, moisture molecules begin to migrate from the surface of the material inward. This movement progresses to the interior of the material over time, gradually increasing the material’s moisture content.

From Fick’s second law of diffusion, Equation (4) describes moisture uptake in composite materials, while Equation (5) expresses the mass change due to moisture uptake.

(4) $$D=\pi {\left(\frac{h}{4M_{\infty }}\right)}^{2}{\left(\frac{M_{2}-M_{1}}{{\sqrt{t}}_{2}-\sqrt{t_{1}}}\right)}^{2}{\left(1+\frac{h}{L}+\frac{h}{w}\right)}^{-2}$$

(5) $$M=\left[1-\frac{8}{\pi^{2}}\exp\left(-\pi^{2}\frac{D_{t}}{h^{2}}\right)\right]M\infty$$

where

D: moisture uptake,
M: mass change,
M_1_ and M_2_: the moisture contents,
t_1_ and t_2_: time,
h: composite thickness,
L: composite length,
W: composite width,
M∞: maximum change in mass

The moisture absorption capacity of GFRP adhesive joints is higher than that of CFRP. This is due to the glass fibers being more permeable to water molecules and their interaction with the epoxy matrix [19]. An increase in moisture retention over time was also observed in CFRP specimens; however, this increase was much less than in GFRP. This difference is due to the lower water absorption tendency and more microstructure of carbon fibers [34].

Table 4 below shows the average moisture retention rates (%) of GFRP and CFRP connections in offshore wind turbine blade connections measured after 1, 2, and 3 months of seawater immersion. For both materials, moisture absorption reached saturation, particularly from month 3 onward, and no significant increase in moisture retention rates was observed. This suggests that the water uptake kinetics of the materials slowed over time and that the material matrix and fiber-matrix interface became more stable. Therefore, the moisture retention values measured after month 3 represent the maximum moisture absorption capacity of the material under long-term seawater exposure.

According to Table 4:

- GFRP joint specimens absorbed more moisture compared to CFRP. This increased moisture uptake leads to mechanical weakening and interfacial degradation at the structural joints in GFRP.
- While moisture absorption in GFRP increases significantly over time, the increase in CFRP is comparatively lower.
- This indicates that CFRP offers better environmental durability in structural composite applications operating in humid and salty environments, such as offshore wind turbine blade joints.

### 3.2. Four Point Bending Test Results

#### GFRP and CFRP Specimen Details

- Reference Samples—Dry Conditioned (GFRP-7L-FPBT-DE-1S and CFRP-8L-FPBT-DE-1S)

**Figure 11:**
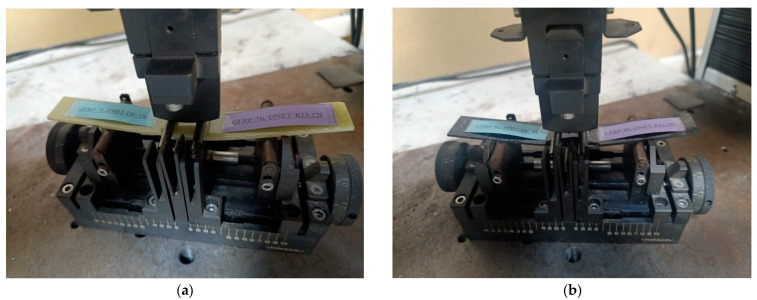
Four-point bending tester and positioning of GFRP (**a**) and CFRP (**b**) dry specimens.

The placement of GFRP and CFRP joint specimens, which were kept in a dry environment, in a four-point bending test apparatus and the test setup are presented in Figure 11. This test setup is designed to evaluate the mechanical behavior of composite structures under bending. The specimens are supported at two end points, and forces are applied through two equally spaced loading points. This ensures a homogeneous stress distribution in the central constant moment region [31].

In the test setup, the bonded joint region is positioned to coincide with the central constant moment region. This allows for the observation of damage occurring at the joint interface. Figure 11 shows in detail the support points, loading pins, and the direction of the test, which ensure the correct alignment of the specimen.

**Figure 12:**
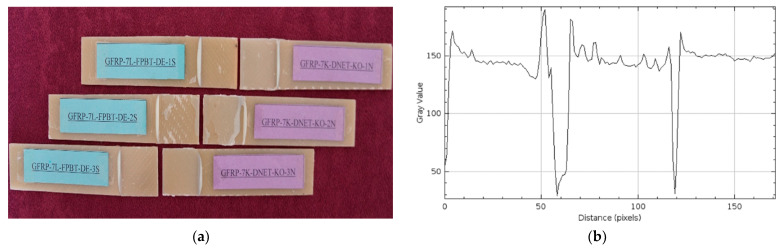
GFRP-7L-FPBT-DE Dry Conditioned (**a**) and surface roughness profile along the damage zone (**b**).

**Figure 13:**
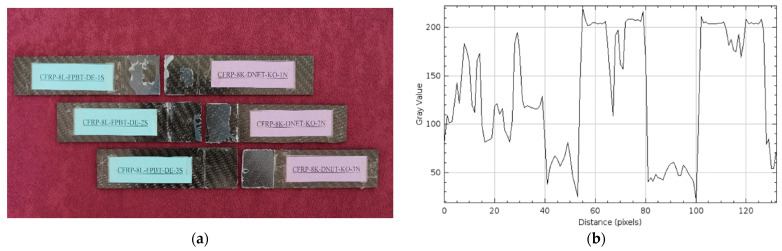
CFRP-8L-FPBT-DE Dry Conditioned (**a**) and surface roughness profile along the damage zone (**b**).

Damage observations after four-point bending of GFRP and CFRP reference samples stored in a dry environment at 21 °C for three different samples are given in Figure 12 and Figure 13, respectively. These samples were used as references for comparative evaluation with samples exposed to seawater for 1, 2, and 3 months.

In Figure 12a, separations were observed in all GFRP specimens starting from the bond line. These separations were caused by the maximum bending stress occurring in the constant moment region between the two points where the loads were applied in the four-point bending test. Damage was concentrated in the bending region midway between the load application points, and the integrity of the specimen body was largely preserved. This suggests that rupture behavior began in this region, where the highest bending stress was effective.

Figure 13a shows the CFRP specimens after the four-point bending test. In the CFRP specimens, separation occurred at the bond line, particularly between the load application points, and the integrity of the specimens was maintained. Figure 13 shows that the damage at the bond line began in the constant moment region in the middle, where the maximum bending stress was effective, and progressed along the bond line.

Comparing the surface roughness profile along the damage area in Figure 12b and Figure 13b:

In Figure 12b, the maximum gray value of the surface profile of the GFRP specimen was measured as approximately 150, and the distance (pixels) value was measured as 150. This indicates a regular but slightly rough surface structure. Despite being conditioned in a dry environment, the damage type, such as limited micro-level distortion, was observed at the fiber-matrix interface after the four-point bending test, resulting in the profile exhibiting wide-ranging but low-intensity fluctuations.

In the CFRP sample in Figure 13b, the maximum gray value exceeds 200, while the distance (pixels) value remains around 120. This profile demonstrates more intense and distinct structural features on the CFRP surface, indicating a more rigid fiber-matrix interface. A higher gray value indicates a surface feature where the fibers form a stronger bond with the matrix, while a shorter distance indicates that this integrity is maintained in a more localized yet robust structure.

- One-Month Sea Water Exposure Samples (GFRP-7L-FPBT-1M and CFRP-8L-FPBT-1M)

**Figure 14:**
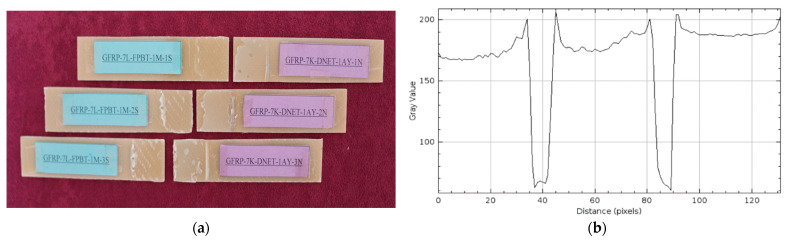
GFRP-7L-FPBT-1M Samples Kept In Seawater For 1 Month (**a**) and surface roughness profile along the damage zone (**b**).

**Figure 15:**
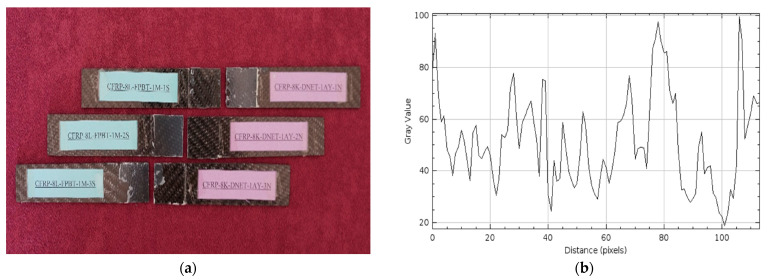
CFRP-8L-FPBT-1M Samples Kept In Seawater For 1 Month (**a**) and surface roughness profile along the damage zone (**b**).

The damage types that occurred after the four-point bending tests performed on GFRP (a) and CFRP (b) samples kept in sea water at a constant temperature of 21 °C for 1 month are presented in Figure 14 and Figure 15, respectively, in three different samples.

The samples in Figure 14a were weakened chemically and physically by seawater penetrating the bond interface through the sample. The diffusion of ions in the seawater began to disrupt the bond integrity between the adhesive and the composite samples, reducing the adhesion quality at the interface.

The sudden rupture behavior observed in the CFRP samples in Figure 15a is due to the high structural strength of the carbon fibers. Due to their low water absorption, the CFRP samples were able to maintain adhesion at the bond line for longer periods and exhibited sudden, but higher, fracture capacity under four-point bending loading.

In the GFRP sample in Figure 14b, the maximum gray value of the surface profile reached 200, and the distance value was measured at approximately 120. These data indicate that damage such as significant microstructural deterioration, fiber shrinkage, and matrix separation occurred on the GFRP surface under the influence of seawater. The increase in the gray value compared to the dry environment reveals that the surface irregularities are more concentrated. The distance value of around 120 indicates that this damage effectively spreads to a specific location.

In the CFRP sample in Figure 15b, the maximum gray value was only slightly above 100, while the distance value was measured at approximately 100. A low gray value indicates that surface irregularities are limited and damage is less severe. A low distance value, however, indicates that damage occurs in a narrower, more limited area. This indicates that CFRP specimens maintain their structural integrity better in the seawater environment than GFRP specimens, with less decomposition and deterioration occurring at the fiber-matrix interface.

- Two-Month Seawater Exposure Samples (GFRP-7L-FPBT-2M and CFRP-8L-FPBT-2M)

**Figure 16:**
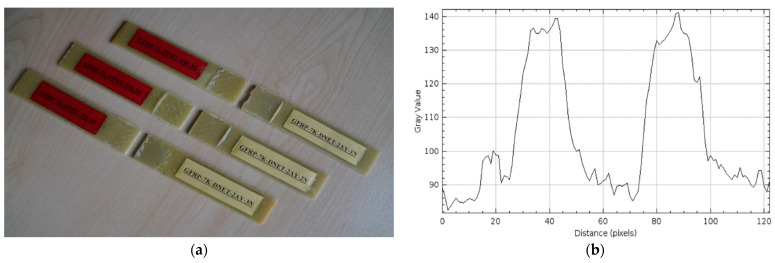
GFRP-7L-FPBT-2M Samples Were Kept In Sea Water For 2 Months (**a**) and surface roughness profile along the damage zone (**b**).

**Figure 17:**
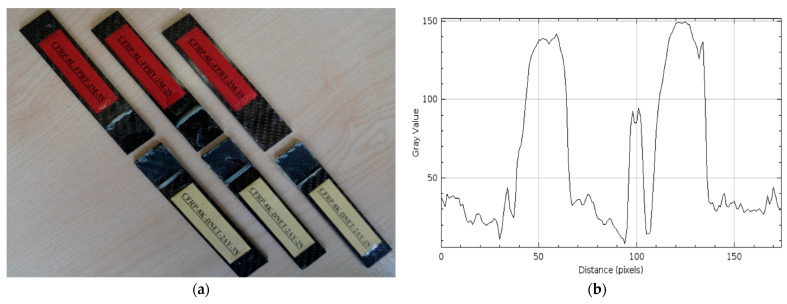
CFRP-8L-FPBT-2M Samples Were Kept In Sea Water For 2 Months (**a**) and surface roughness profile along the damage zone (**b**).

The damage types that occurred after the four-point bending tests performed on GFRP (a) and CFRP (b) samples kept in sea water at a constant temperature of 21 °C for 2 months are presented in Figure 16 and Figure 17, respectively, in three different samples.

In the samples shown in Figure 16a, a mixed (cohesive + adhesive) type of damage was observed, where the damage was not confined to the bond line, but adhesive residue was also present on both surfaces, and both interfacial and intra-adhesive fractures occurred simultaneously. This indicated that during loading, both the internal structure of the adhesive in the bond region weakened and the bond interfaces separated. This distribution of damage indicated that the stress distribution at the bond line was uneven, and seawater weakened the bond.

Similarly, in Figure 17a, the presence of adhesive residue on both surfaces along with the rupture at the adhesive bond line in the CFRP samples indicates a mixed (cohesive + adhesive) damage type. Despite 2 months of immersion in seawater, CFRP’s superior mechanical properties ensured that the loads transferred from the adhesive to the material were more evenly distributed at the interface, resulting in better adhesion of the adhesive to the sample surface.

In Figure 16b, the maximum gray value and distance (pixels) values for the GFRP samples were measured at approximately 140 and 120, respectively. A decrease in the gray value compared to the one-month post-treatment period indicates that surface differences and microstructural damage have diminished. However, the fact that the distance value remained the same indicates that the spread of surface damage continues at a similar rate.

In Figure 17b, the maximum gray value and distance values for the CFRP samples were measured at 150 and 150, respectively. Compared to the one-month post-treatment period, an increase in the gray value and distance values was observed. This is because the damage in CFRP samples increases over time.

- Three-Month Seawater Exposure Samples (GFRP-7L-FPBT-3M and CFRP-8L-FPBT-3M)

**Figure 18:**
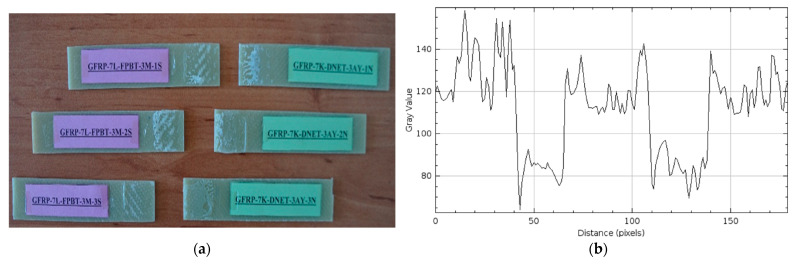
GFRP-7L-FPBT-3M Samples Were Kept in Sea Water for 3 Months (**a**) and surface roughness profile along the damage zone (**b**).

**Figure 19:**
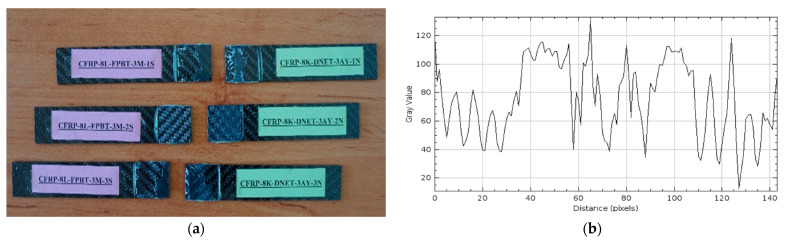
CFRP-8L-FPBT-3M Samples Were Kept in Sea Water for 3 Months (**a**) and surface roughness profile along the damage zone (**b**).

The damage types that occurred after four-point bending tests performed on GFRP (a) and CFRP (b) samples kept in seawater at a constant temperature of 21 °C for 3 months are presented in Figure 18 and Figure 19, respectively, in three different samples.

In the GFRP specimens exposed to seawater for 3 months, shown in Figure 18a, damage was observed to occur directly at the bond line. The failure was particularly concentrated in the central region of the joint, and adhesive residue remained on both surfaces of the specimens. This indicates that the damage was predominantly adhesive, weakening the bond strength at the bond surface. Moisture penetrating the adhesive-sample interface during seawater exposure reduced adhesion quality and created microscopic voids and a plasticizing effect at the interface. This weakened the bond line.

In Figure 19a, similar damage occurred at the bond line in CFRP samples exposed to seawater for 3 months, with the adhesive remaining on both surfaces, resulting in a cohesive-adhesive transition. However, given the high strength and low moisture absorption capacity of CFRP samples, the damage is less severe compared to GFRP samples.

In Figure 18b, the maximum gray value for the GFRP samples exceeded 140, and the distance value was measured as 150 pixels. Compared to 2 months, there was no significant increase in the gray value, but the distance increased. This indicates that the roughness on the GFRP surface began to remain constant, but the deterioration spread over a wider area. In other words, the GFRP samples continued to absorb seawater, leading to microstructural deterioration on the surface.

In Figure 19b, the maximum gray value for the CFRP samples was determined as 120 and the distance value as 140. Compared to the two-month period, the gray value decreased, but the distance value increased. This change indicates that the roughness of the CFRP surface has decreased somewhat, but the deterioration has spread over a larger surface area. This suggests that although the CFRP samples were initially resistant to seawater conditions, they began to struggle to maintain their surface integrity after prolonged exposure. In Table 5 below, the average surface roughness (Ra) values of GFRP and CFRP composite samples are given.

The surface roughness values in Table 5 are a decisive factor in indicating the surface area in contact with the adhesive and, therefore, the state of mechanical bonding.

Accordingly, the highest roughness value, GFRP-7L-FPBT-1M = 98.00 µm, indicates significant surface deterioration and matrix separation in the GFRP composite joint after 1 month of exposure.

The lowest roughness value, CFRP-8L-FPBT-1M = 25.00 µm, indicates that the CFRP composite joint creates a smoother surface due to the smoother fracture of the fiber structure.

Roughness values for both connection samples at 2 and 3 months of exposure (GFRP-7L-FPBT-2M = 58.95 µm, GFRP-7L-FPBT-3M = 67.24 µm) (CFRP-8L-FPBT-2M = 41.22 µm, CFRP-8L-FPBT-3M = 34.48 µm) were at moderate levels. This indicates that the surface area in contact with the adhesive was sufficient and provided a suitable roughness for mechanical bonding. Considering these values, GFRP surfaces, with their higher roughness, have stronger mechanical interlocking potential, while CFRP surfaces, with their smoother surfaces, can provide more controlled load transfer with the adhesive.

GFRP’s higher roughness provides greater mechanical interlocking between the adhesive and the surface. This generally results in higher bond strength and a stronger bond. However, excessive roughness creates voids or stress concentrations in the adhesive, leading to long-term weakening.

CFRP’s smoother surface allows the adhesive layer to distribute stress more uniformly and more controllably. This creates a more durable and stable joint, especially in high-performance applications.

**Figure 20:**
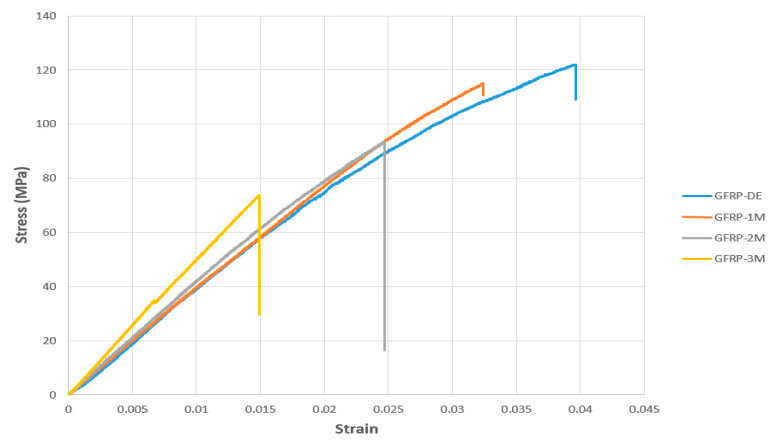
Comparison of Stress–Strain Graphs of GFRP Specimens Stored in Dry and Seawater Environments (1, 2 and 3 months).

The stress–strain (σ–ε) curve presented in Figure 20 shows data obtained from four-point bending tests performed on GFRP composite specimens after exposure to various environmental conditions for specific periods. The specimens were stored in dry environments and in seawater environments for 1, 2, and 3 months, respectively. The effects of these different conditions and exposure times on the mechanical properties of the material were analyzed in detail.

Based on the data presented in Figure 20, it was observed that the bending stress and strain values in the GFRP-7L-FPBT series specimens decreased depending on the duration of exposure to seawater. The measurement results for each specimen are presented in Table 6 below:

The flexural stress and strain values of the GFRP-7L-FPBT series specimens presented in Figure 20 indicate a significant deterioration in the mechanical properties of the material depending on the duration of seawater exposure. The reference specimen tested under dry conditions (GFRP-7L-FPBT-DE) exhibited the highest mechanical performance with a flexural stress of 121.6930 MPa and a strain value of 0.0395.

For the GFRP-7L-FPBT-1M specimen, which was exposed to seawater for one month, the flexural stress dropped to 114.9519 MPa and the strain value to 0.0323. This reduction suggests that seawater penetrated the GFRP matrix, leading to weakening at the fiber-matrix interface.

At the end of the second month (GFRP-7L-FPBT-2M), the flexural stress was measured at 92.6155 MPa and the strain at 0.0244. By the third month (GFRP-7L-FPBT-3M), these values further decreased to 72.7945 MPa and 0.0146, respectively, indicating a decline in both mechanical strength and deformation capacity.

Overall, as the seawater exposure duration increased, both flexural stress and strain values consistently decreased in GFRP specimens. With a total reduction of approximately 40% in flexural stress and up to 63% in strain, these results suggest that GFRP may exhibit limited long-term mechanical performance in marine environments.

**Figure 21:**
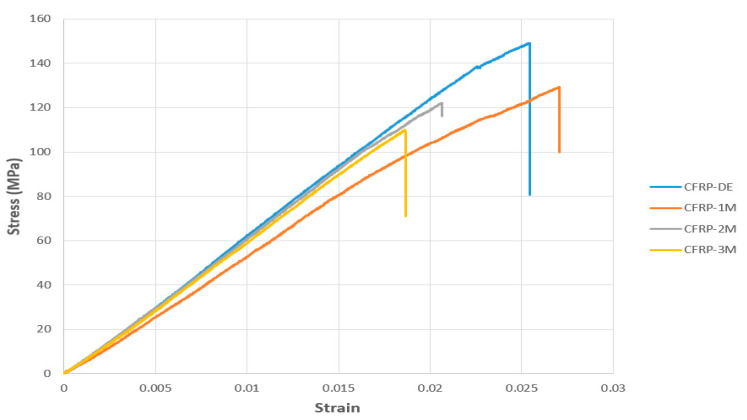
Comparison of Stress–Strain Graphs of CFRP Specimens Stored in Dry and Seawater Environments (1, 2 and 3 Months).

According to the data presented in Figure 21, it was observed that the bending stress and strain values in the CFRP-8L-FPBT series specimens varied depending on the duration of exposure to seawater. Table 7 below shows the measurement results for each specimen:

The bending stress and strain values for the CFRP-8L-FPBT series specimens, presented in Figure 21, reveal a gradual decrease in the material’s mechanical properties with the duration of exposure to seawater. The reference specimen (CFRP-8L-FPBT-DE), tested in a dry environment, achieved the highest performance with a bending stress of 148.5722 MPa and a strain of 0.0254.

In the CFRP-8L-FPBT-1M specimen exposed to seawater for one month, the bending stress decreased to 129.2385 MPa, while the strain value increased slightly to 0.0270. This increase was attributed to the short-term softening effect of moisture, which imparted temporary flexibility to the matrix phase.

In the second month, both the bending stress (121.9446 MPa) and strain (0.0206) decreased in the CFRP-8L-FPBT-2M specimen. At the end of the third month (CFRP-8L-FPBT-3M), the bending stress decreased to 109.5578 MPa, indicating an approximately 26% decrease compared to the initial value. At the same time, the strain value decreased to 0.0185, indicating a significant decrease in the material’s deformation capacity. Consequently, a continuous decrease in the bending stress and strain values of the CFRP samples was observed as the duration of seawater exposure increased. This demonstrates that the aging process negatively affects the samples in terms of both strength and ductility. However, this decrease was less pronounced compared to the GFRP samples. This demonstrates that CFRP samples are more durable in applications with intense environmental impacts, such as offshore wind turbine blades.

Young’s Modulus is a parameter that numerically expresses the flexibility or stiffness of a material [35]. Here, it was calculated using the slope obtained from the linear regions of the stress–strain curves presented in Figure 20 and Figure 21. Table 7 below presents a comparison of the modulus of elasticity (Young’s Modulus, E) values of GFRP and CFRP specimens. These values were calculated after the respective specimens were exposed to environmental conditions for different periods of time. Table 8 also shows the percentage change in modulus of elasticity for each specimen compared to its initial (dry environment) value.

### 3.3. Damage Investigation of GFRP Specimens Using SEM After Four-Point Bending Test

As exposure to seawater increases, the types of damage occurring in the interlayer separation and joint areas of the composite samples become more pronounced. Many factors, including the material’s microstructure, joint configuration, and the properties of the adhesive used, play a role in the formation of these damages. Therefore, detailed characterization of such composite structures is critical.

**Figure 22:**
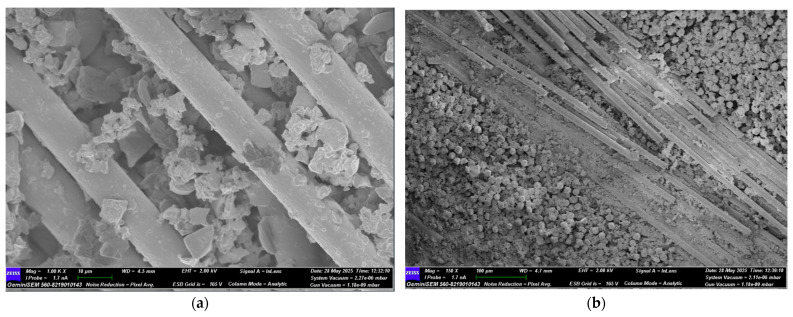
(**a**) Mag = 1.00 KX and (**b**) Mag = 150X Images of GFRP Samples not kept in sea water.

**Figure 23:**
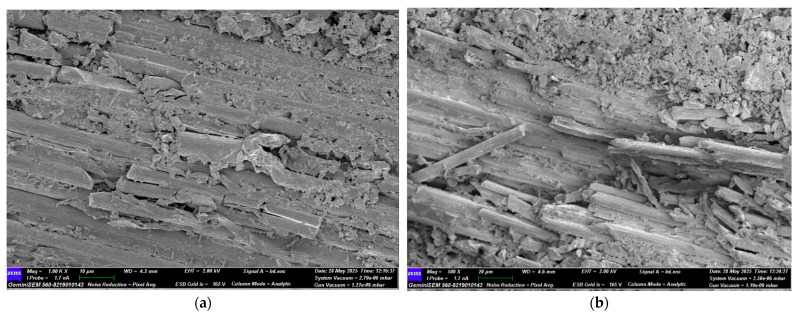
(**a**) Mag = 1.00 KX and (**b**) Mag = 500X Images of GFRP Samples 1st Month.

**Figure 24:**
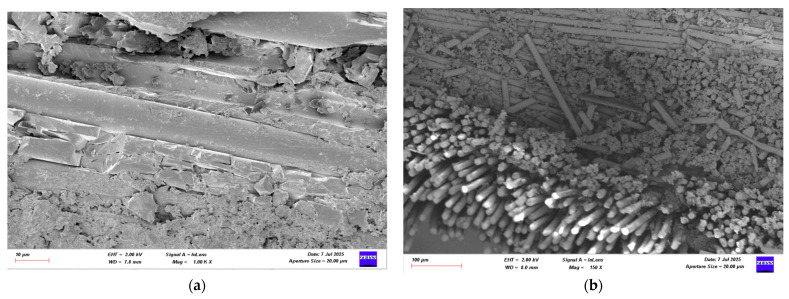
(**a**) Mag = 1.00 KX and (**b**) Mag = 150X Images of GFRP Samples 2nd Month.

**Figure 25:**
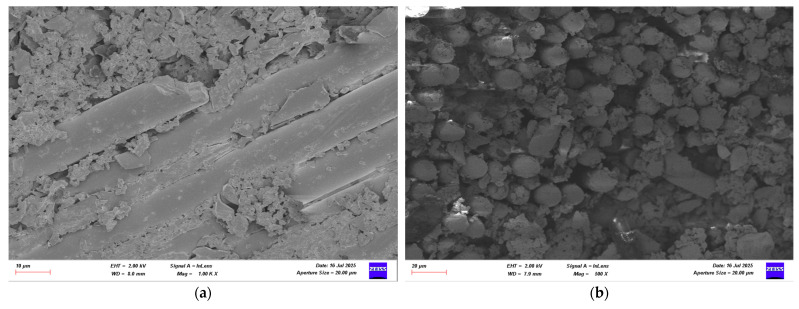
(**a**) Mag = 1.00 KX and (**b**) Mag = 500X Images of GFRP Samples 3rd Month.

**Figure 26:**
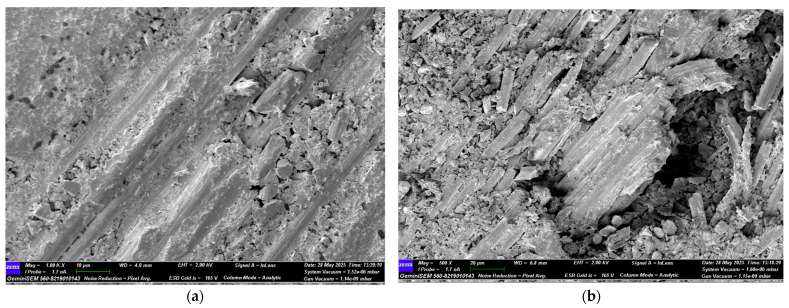
(**a**) Mag = 1.00 KX and (**b**) Mag = 500X Images of CFRP Samples not kept in sea water.

**Figure 27:**
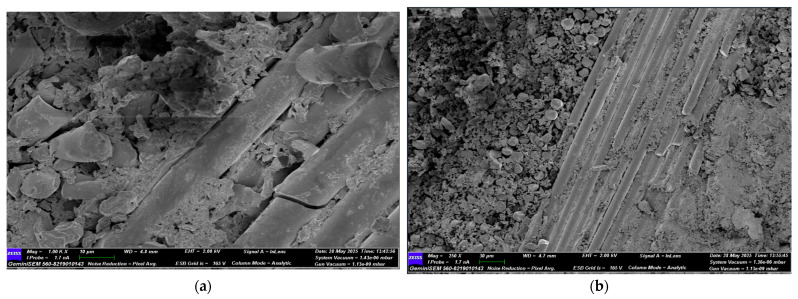
(**a**) Mag = 1.00 KX and (**b**) Mag = 250X Images of CFRP Samples 1st Month.

**Figure 28:**
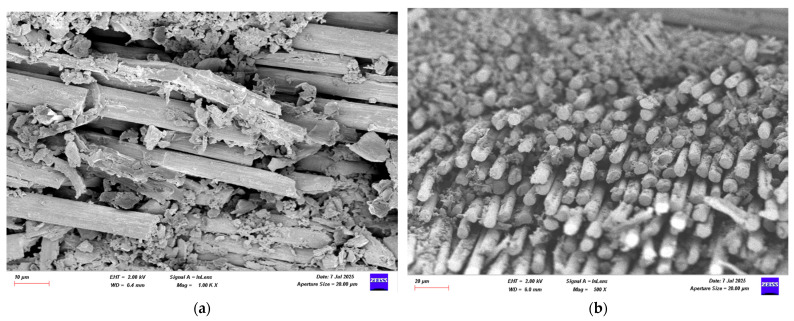
(**a**) Mag = 1.00 KX and (**b**) Mag = 500X Images of CFRP Samples 2nd Month.

**Figure 29:**
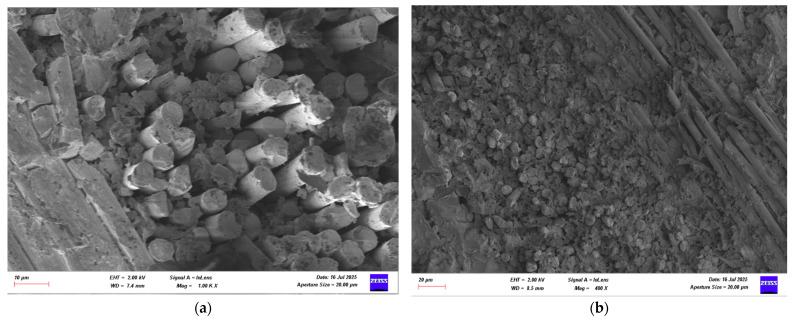
(**a**) Mag = 1.00 KX and (**b**) Mag = 400X Images of CFRP Samples 3rd Month.

Following the four-point bending test, SEM images of GFRP samples stored in seawater for 1, 2, and 3 months were obtained for both dry and offshore conditions. Images of the GFRP samples are presented in Figure 22, Figure 23, Figure 24 and Figure 25. Images of the CFRP samples are presented in Figure 26, Figure 27, Figure 28 and Figure 29. Thus, the time-dependent changes in damage mechanisms in samples exposed to environmental influences were comparatively evaluated. Following the four-point bending test, SEM images were obtained for both GFRP and CFRP specimens that had been exposed to seawater for 1, 2, and 3 months, as well as those kept under dry and offshore conditions. In this way, time-dependent changes in the damage mechanisms of the seawater-exposed specimens were comparatively evaluated. The SEM images of the GFRP specimens are presented in Figure 22, Figure 23, Figure 24 and Figure 25.

In Figure 22, SEM images of the GFRP control sample stored in a dry environment show strong interfacial bonding with the polymer matrix around the glass fibers. There is a regular arrangement between the fibers, minimal voids, and continuity in the fiber-matrix interaction. No extensive fractures or bifurcations are observed on the fibers, indicating that the sample maintains its high flexural strength.

In Figure 23, in GFRP samples exposed to seawater for one month, deterioration began at the fiber-matrix interface, resulting in damage mechanisms such as debonding and fiber pull-out. Microcracks and traces of plastic deformation were observed on the matrix surface. Diffusion of water molecules into the matrix caused the matrix to swell and accumulate stress at the interface, weakening the bond. Mechanical strength decreased compared to the dry environment, but integrity was still partially preserved. Figure 24 shows significant microstructural deterioration in the GFRP samples following two months of seawater storage. While fracture and shrinkage were observed in the majority of the fibers, the matrix exhibited intense microcracks, fragmentation, and void formation. The interface exhibited completely ruptured areas, and the fiber-matrix interaction was largely lost. At this stage, load transfer occurred only to a limited extent through the fibers. A decrease in flexural strength, a tendency towards fracture, and brittleness were observed. Figure 25 shows almost complete loss of microstructural integrity in GFRP specimens conditioned in seawater for three months. A structure in which the fiber-matrix interface was reduced, the fibers became free, and the matrix was chemically degraded is envisaged. The microscopic cracks transformed into macroscopic cracks, and the fracture occurred suddenly and brittle. Furthermore, the mechanical strength of the polymer matrix decreased as a result of exposure to water.

The effect of seawater conditioning on the microstructural integrity of GFRP specimens is given in Table 9.

### 3.4. Damage Investigation of CFRP Specimens Using SEM After Four-Point Bending Test

Following the four-point bending test, SEM images of CFRP specimens conditioned in seawater for 1, 2, and 3 months were obtained for both dry and offshore conditions. Images of the CFRP specimens are presented in Figure 26, Figure 27, Figure 28 and Figure 29.

Figure 26, the CFRP control sample stored in a dry environment, shows that the fiber-matrix interface has high integrity. The fibers are tightly bonded to the matrix and exhibit a regular structure along the fracture surface. Fiber pullout and fiber fracture appear to be balanced. This indicates that energy was absorbed more ductilely during mechanical testing and that fracture occurred in a controlled manner. Figure 27 shows that signs of microstructural deterioration began to appear in samples stored in seawater for one month. Separation occurred locally at the fiber-matrix interface, with microscopic cracks and matrix deformations observed. This indicates that the interfacial bonds began to weaken and that mechanical performance tended to decline. Figure 28 shows that after two months of aging, microstructural deterioration increased significantly. It was observed that the fibers began to completely separate from the matrix. Swelling, cracking, and void formation are evident in the resin phase. Fiber fractures are more irregular and short-distanced, and the fracture surface exhibits a more brittle state. In Figure 29, microstructural deterioration progressed in samples exposed to seawater for three months, and interfacial bonds became loose. Delamination and voids were observed in the resin structure, leading to a decrease in mechanical performance.

The effect of seawater conditioning on the microstructural integrity of CFRP specimens is given in Table 10.

SEM images and mechanical test data obtained after immersing GFRP and CFRP composite samples in seawater for 1, 2 and 3 months, both in dry environments and offshore conditions, reveal that both composite types lose their structural integrity over time. Significant decomposition was observed, particularly at the fiber-matrix interface, with increasing aging time. Damage mechanisms such as fiber pullout, debonding, microscopic cracks, and matrix deformations increased over time in both GFRP and CFRP samples exposed to seawater. Comparisons revealed that CFRP composite samples exhibited higher microstructural resistance to seawater compared to GFRP. Because the chemical and mechanical bond between carbon fibers and epoxy resin is stronger than that of glass fibers, the interface bonds weakened more slowly in CFRP samples, and the fracture transition was more controlled [36]. While microcracks were observed in both samples during the dry and first months, these cracks coalesced over time, transforming into macroscopic delaminations and delamination. At the end of the second and third months, signs of degradation and swelling in the chemical structure of the polymer matrix were clearly observed, particularly in the GFRP samples. With increasing aging, a greater decrease in flexural strength and interfacial strength was observed in the GFRP samples compared to the CFRP samples. The CFRP composite samples were found to be more resistant to these aging conditions compared to GFRP due to their better interface and lower water permeability.

## 4. Discussion and Conclusions

This study investigated the durability of adhesively bonded GFRP and CFRP joints under marine conditions and specifically evaluated the performance of composite materials used in offshore wind turbine blades. Seven-layer GFRP and eight-layer CFRP laminates were prepared in accordance with ASTM standards, and specimens were exposed to natural Aegean Sea water (21 °C, salinity 3.3–3.7%) for 1, 2, and 3 months. Measurements showed that GFRP’s moisture absorption capacity was significantly higher than CFRP’s (GFRP: from 1.02% to 3.78%; CFRP: from 0.49% to 0.91%). Four-point bending tests conducted according to ASTM D790 revealed a 9.45% decrease in Young’s modulus for GFRP and a 3.48% decrease for CFRP. SEM images revealed that delamination, matrix cracking, and adhesion loss were more intense in GFRP joints under the influence of seawater. Despite relatively low moisture absorption, brittle fiber ruptures and interface separations were observed in CFRP joints. These findings confirm that environmental exposure reduces mechanical performance by accelerating damage mechanisms, particularly in bond regions. The F-RES 21 epoxy resin and F-Hard 22 hardener used contributed to maintaining structural integrity in both material groups, but CFRP specimens demonstrated higher flexural strength even in seawater conditions.

Consequently, CFRP connections were determined to exhibit superior mechanical strength and structural stability compared to GFRP in long-term marine environments.

This study highlights the importance of material selection and environmental influences on the durability of composite elements operating in harsh environments such as offshore structures, and demonstrates the importance of connection design and structural integrity, along with experimentally investigated stress distribution and damage propagation mechanisms, in developing reliable designs.

## Figures and Tables

**Table 1:** Coding system for identifying GFRP and CFRP specimens.

Code (English)	Description
GFRP-7L-FPBT-2M-1S	7-layer GFRP, four-point bending test, 2 months in seawater, specimen no. 1
CFRP-8L-FPBT-3M-2S	8-layer CFRP, four-point bending test, 3 months in seawater, specimen no. 2

**Table 2:** Moisture Retention Rates (%) of GFRP Specimens Exposed to Seawater for Different Durations.

Dry Sample Code	Dry Weight (g)	Store Time	Soaked in Sea Water Sample Code	Wet Weight (g)	Moisture Retention Rate (%)
GFRP-7L-FPBT-DE	17.2374	1st Month	GFRP-7L-FPBT-1M-1S	17.3524	1.02
GFRP-7L-FPBT-DE	17.2374	2nd Month	GFRP-7L-FPBT-2M-2S	17.8593	2.97
GFRP-7L-FPBT-DE	17.2374	3rd Month	GFRP-7L-FPBT-3M-3S	17.9707	3.78

**Table 3:** Moisture Retention Rates (%) of CFRP Specimens Exposed to Seawater for Different Durations.

Dry Sample Code	Dry Weight (g)	Store Time	Soaked in Sea Water Sample Code	Wet Weight (g)	Moisture Retention Rate (%)
CFRP-8L-FPBT-DE	14.7695	1st Month	CFRP-8L-FPBT-1M-1S	14.8808	0.49
CFRP-8L-FPBT-DE	14.7695	2nd Month	CFRP-8L-FPBT-2M-2S	14.9247	0.76
CFRP-8L-FPBT-DE	14.7695	3rd Month	CFRP-8L-FPBT-3M-3S	14.9252	0.91

**Table 4:** Comparison of Moisture Retention Rates.

Exposure Time (Months)	GFRP Moisture Absorption Rate (%)	CFRP Moisture Absorption Rate (%)
1	1.02	0.49
2	2.97	0.76
3	3.78	0.91

**Table 5:** Surface roughness values for GFRP and CFRP samples.

Specimen Code	Average Roughness, Ra (µm)
GFRP-7L-FPBT-DE	71.81
CFRP-8L-FPBT-DE	67.05
GFRP-7L-FPBT-1M	98.00
CFRP-8L-FPBT-1M	25.00
GFRP-7L-FPBT-2M	58.95
CFRP-8L-FPBT-2M	41.22
GFRP-7L-FPBT-3M	67.24
CFRP-8L-FPBT-3M	34.48

**Table 6:** Mechanical Properties of GFRP-7L-FPBT Series Specimens (Figure 20 Data).

Specimen Code	Flexural Stress (MPa)	Average (Flexural Stress)	Std. Dev. (Flexural Stress)	Strain (ε)	Average (Strain)	Std. Dev. (Strain)	Seawater Exposure Duration
GFRP-7L-FPBT-DE	121.693	100.263	22.28	0.0395	0.0277	0.0107	None (Dry Condition)
GFRP-7L-FPBT-1M	114.951			0.0323			1 Month
GFRP-7L-FPBT-2M	92.615			0.0244			2 Months
GFRP-7L-FPBT-3M	72.794			0.0146			3 Months

**Table 7:** Mechanical Properties of CFRP-8L-FPBT Series Specimens (Figure 21 Data).

Specimen Code	Flexural Stress (MPa)	Average (Flexural Stress)	Std. Dev. (Flexural Stress)	Strain (ε)	Average (Strain)	Std. Dev. (Strain)	Seawater Exposure Duration
CFRP-8L-FPBT-DE	148.572	125.615	16.43	0.0270	0.0229	0.0040	None (Dry Condition)
CFRP-8L-FPBT-1M	122.385			0.0254			1 Month
CFRP-8L-FPBT-2M	121.944			0.0206			2 Months
CFRP-8L-FPBT-3M	109.557			0.0185			3 Months

**Table 8:** Comparison of Young’s Modulus (E) for GFRP and CFRP Specimens.

Specimen Code	Elastic Modulus (MPa)	Average	Std. Dev.	Material Type	Change Compared to Initial (%)
CFRP-8L-FPBT-DE	6.270	6.154	0.096	CFRP	Reference
CFRP-8L-FPBT-1M	6.189			CFRP	1.29
CFRP-8L-FPBT-2M	6.106			CFRP	2.62
CFRP-8L-FPBT-3M	6.052			CFRP	3.48
GFRP-7L-FPBT-DE	3.878	3.697	0.156	GFRP	Reference
GFRP-7L-FPBT-1M	3.756			GFRP	3.15
GFRP-7L-FPBT-2M	3.644			GFRP	6.42
GFRP-7L-FPBT-3M	3.510			GFRP	9.45

**Table 9:** Effect of Seawater Conditioning on the Microstructural Integrity of GFRP Specimens.

Figure No	Condition	Fiber-Matrix Interface	Observed Damage Mechanisms	Microstructural Condition	Mechanical Strength
Figure 22	Dry Condition	Strong bonding	Fiber pull-out	Regular fiber alignment, minimal voids	High
Figure 23	1 Month Seawater	Initial degradation	Debonding, fiber pull-out, microcracks	Matrix swelling, weakened bonding	Reduced
Figure 24	2 Months Seawater	Significant damage	Fiber breakage, matrix fragmentation, voids	Loss of fiber-matrix interaction	Reduced
Figure 25	3 Months Seawater	Almost completely degraded	Macrocracks, chemical matrix degradation	Fibers free, merged cracks, brittle failure	Low

**Table 10:** Effect of Seawater Conditioning on the Microstructural Integrity of CFRP Specimens.

Figure No	Condition	Fiber-Matrix Interface	Observed Damage Mechanisms	Microstructural Condition	Mechanical Strength
Figure 26	Dry Condition	High integrity	Balanced fiber breakage	Regular structure, controlled fracture	High
Figure 27	1 Month Seawater	Partial debonding	Microcracks, matrix deformation	Initial degradation, weakened bonding	Reduced
Figure 28	2 Months Seawater	Severe degradation	Fiber-matrix separation, resin cracking and voids	Brittle fracture, irregular fiber breakage	Lower
Figure 29	3 Months Seawater	Interface detached	Delamination, void formation	Free fibers	Very low

## Data Availability

The original contributions presented in the study are included in the article, further inquiries can be directed to the corresponding author.
